# Letter from the Editor in Chief

**DOI:** 10.19102/icrm.2025.16105

**Published:** 2025-10-15

**Authors:** Devi Nair


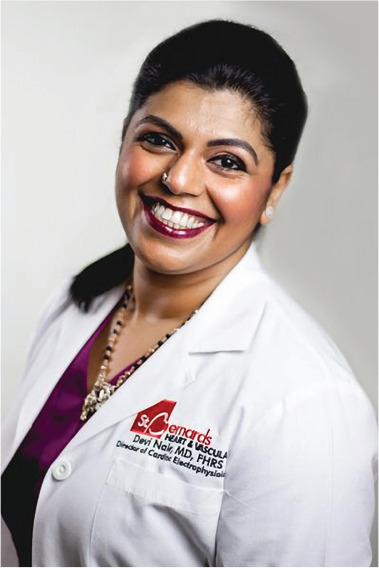


Dear Colleagues,

October has been a month that reaffirmed both the science and the spirit of our specialty. Physiologic pacing is no longer the future—it is the present. The recent 9th Annual International Physiology of Pacing Symposium showcased how our field continues to evolve—uniting anatomy, technology, and education to refine how we restore the heart’s natural activation.

## The Physiology of Pacing: A Global Conversation

This year’s symposium brought together world-renowned experts and an international audience for a comprehensive exploration of conduction system pacing. The program featured in-depth, case-based discussions on His-bundle pacing, left bundle branch area pacing (LBBAP), and left ventricular septal pacing—each representing a major step toward restoring physiologic, efficient electrical activation.

Attendees left with not only practical skills but also a deeper understanding of how these strategies are transforming care for patients with heart failure, atrioventricular block, and complex bradyarrhythmias. The symposium underscored a vital truth: physiologic pacing is both a technological and conceptual shift—embracing the heart’s own conduction system as the optimal resynchronization pathway.

As we reflect on the symposium, it is fitting that most of this month’s manuscripts also focus on pacing—each exploring innovation, precision, and clinical impact within this transformative field.

## Advances in Science and Clinical Practice

This issue highlights how technology and insight continue to advance pacing therapy and rhythm management:

- ***Three-dimensional mapping for left bundle branch area pacing.*** Niazi and colleagues^1^ introduce an innovative use of the Navik 3D mapping system to guide LBBAP lead placement using standard fluoroscopy. By reconstructing the interventricular septum in three dimensions, this approach enhances accuracy, reduces fluoroscopy time, and makes physiologic pacing more accessible.
- ***Impact of coronavirus disease 2019 on sick sinus syndrome and pacemaker implantation.*** Dulal et al.^2^ report a significant post-pandemic rise in sick sinus syndrome and pacemaker implantation, particularly among older adults. Their large-scale analysis highlights shifting electrophysiologic patterns following systemic illness and emphasizes vigilance in recognizing conduction changes after infection.
- ***Totally fluoroless subcutaneous implantable cardioverter-defibrillator implantation.*** Archontakis and colleagues^3^ demonstrate that fluoroless subcutaneous implantable cardioverter-defibrillator implantation offers safety and efficacy comparable to limited fluoroscopy-assisted techniques. Their findings affirm that a completely fluoroscopy-free approach can safely become standard practice—improving procedural safety for patients and healthcare teams without compromising outcomes.
- ***Speckle tracking echocardiography in leadless pacemaker patients***. Clark et al.^4^ evaluate global longitudinal strain (GLS) as an early marker of myocardial dysfunction in pediatric and congenital heart disease patients implanted with the Micra™ leadless pacemaker (Medtronic, Minneapolis, MN, USA). Their findings suggest GLS may detect subtle pacing-related changes before ejection fraction declines, highlighting its potential for early identification of pacemaker-induced cardiomyopathy.

## Connecting Discovery and Education

Across both the symposium and this issue’s manuscripts, a unifying theme emerges: progress in pacing is powered by integration—of imaging and anatomy, of technology and training, and of innovation with clinical judgment.

As conduction system pacing moves from specialized expertise to standard practice, education remains the bridge between discovery and broad clinical adoption. Meetings like the Physiology of Pacing Symposium transform evolving evidence into daily care.

## Looking Ahead

October’s alignment of research and education reflects where our field stands today—innovative, collaborative, and deeply patient-centered. The *Journal* remains proud to highlight these advances, where new ideas rapidly become new standards of care.

We hope this issue inspires your continued pursuit of excellence in physiologic pacing and beyond.

Warm regards,


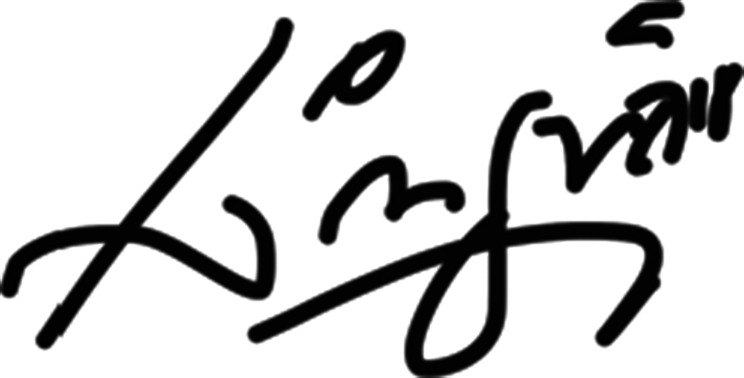


Dr. Devi Nair, md, facc, fhrs

Editor-in-Chief

*The Journal of Innovations in Cardiac Rhythm Management*

Director of the Cardiac Electrophysiology & Research,

St. Bernard’s Heart & Vascular Center, Jonesboro, AR, USA

White River Medical Center, Batesville, AR, USA

President/CEO, Arrhythmia Research Group

Clinical Adjunct Professor, University of Arkansas for Medical Sciences

Governor, Arkansas Chapter of the American College of Cardiology

drdgnair@gmail.com
